# Psycholinguistic and affective norms for 1,252 Spanish idiomatic expressions

**DOI:** 10.1371/journal.pone.0254484

**Published:** 2021-07-16

**Authors:** José M. Gavilán, Juan Haro, José Antonio Hinojosa, Isabel Fraga, Pilar Ferré

**Affiliations:** 1 Department of Psychology, Research Center for Behavior Assessment (CRAMC), Universitat Rovira i Virgili, Tarragona, Spain; 2 Instituto Pluridisciplinar, Universidad Complutense de Madrid, Madrid, Spain; 3 Departamento de Psicología Experimental, Procesos Cognitivos y Logopedia, Universidad Complutense de Madrid, Madrid, Spain; 4 Centro de Ciencia Cognitiva—C3, Universidad Nebrija, Madrid, Spain; 5 Department of Social Psychology, Basic Psychology and Methodology, Cognitive Processes & Behavior Research Group, Universidade de Santiago de Compostela, Santiago de Compostela, Spain; University of Birmingham, UNITED KINGDOM

## Abstract

This study provides psycholinguistic and affective norms for 1,252 Spanish idiomatic expressions. A total of 965 Spanish native speakers rated the idioms in 7 subjective variables: *familiarity*, *knowledge of the expression*, *decomposability*, *literality*, *predictability*, *valence* and *arousal*. Correlational analyses showed that *familiarity* has a strong positive correlation with *knowledge*, suggesting that the knowledge of the figurative meaning of an idiom is highly related to its frequency of use. *Familiarity* has a moderate positive correlation with final word *predictability*, indicating that the more familiar an idiom is rated, the more predictable it tends to be. *Decomposability* shows a moderate positive correlation with *literality*, suggesting that those idioms whose figurative meaning is easier to deduce from their constituents tend to have a plausible literal meaning. In affective terms, Spanish idioms tend to convey more negative (66%) than positive meanings (33%). Furthermore, *valence* and *arousal* show a quadratic relationship, in line with the typical U-shaped relationship found for single words, which means that the more emotionally valenced an idiom is rated, the more arousing it is considered to be. This database will provide researchers with a large pool of stimuli for studying the representation and processing of idioms in healthy and clinical populations.

## Introduction

An idiom is conventionally defined as a trope or figure of speech with a figurative meaning that cannot be deduced from its constituent words [1]. For example, the Spanish idiom “*estirar la pata*” means “*to die*” in figurative terms, but this meaning cannot be derived from the words that make it up: “*estirar*” (stretch), “*la*” (the) or “*pata*” (leg). What distinguishes idioms from other fixed expressions of language is their “non-logical” nature [2]. In other words, for most idioms, there is no logical relationship between their linguistic meaning and their figurative meaning. For example, a syntactic and semantic analysis of the idiom “*estirar la pata*” (kick the bucket) would never lead to the meaning of “dying”. The distance between the literal and figurative meaning of an idiom is related to semantic transparency, which is a property that varies from idiom to idiom. However, the absence of transparency observed in some idioms does not necessarily imply that the relationship between the linguistic and figurative meanings of the idioms is arbitrary.

A number of psycholinguistic studies have investigated how the speakers process idiomatic expressions, leading to different theoretical proposals. For instance, Swinney and Cutler [1] assumed that idioms should be treated like complex words which are represented and understood as whole units. Other authors pointed out that the representation of an idiom depends on the different contributions from the words that make it up and, therefore, when processed, it cannot be understood unless the semantic relationship between its component words is taken into account [3]. Other theoretical proposals, like the hybrid models, assume that idioms can be both compositional (inferred from their component words) and non-compositional. Under the *configuration hypothesis* view [4] the processing of idiomatic expressions depends on familiarity and predictability: the more familiar a sentence is to the listener, the easier it is to recognize a given configuration or idiom. For the *constraint-based model* view [5], idiom processing is affected by different kinds of information (as familiarity, predictability or decomposability) at different points in time.

As can be seen from the above, idioms do not constitute a unitary class and their processing seems to be modulated by several variables. Among them, the most investigated ones have been literality, decomposability, predictability, familiarity and knowledge.

Literality (also called ambiguity, [6]) refers to the degree to which an idiomatic expression has a semantically plausible literal interpretation [7, 8]. Indeed, some idioms have a well-formed literal interpretation, in addition to figurative meaning. For example, “*romper el hielo*” (break the ice) is a highly literal idiom because it can be understood both figuratively (e.g., to start a conversation) and literally (break ice). In contrast, the idiom “*matar el tiempo*” (e.g., do something while you wait; literally: to kill time) does not have a plausible literal interpretation since it is semantically anomalous. Psycholinguistic studies have demonstrated that literality is relevant for idiom processing, being the figurative meaning of idioms with a plausible literal interpretation more accessible than the figurative meaning of idioms without a plausible literal interpretation [5, 9, 10].

Decomposability refers to the extent to which the individual words in an idiomatic expression contribute to its overall idiomatic meaning. For example, the Spanish expression “*no dejar rastro*” (leave no trace) is highly decomposable because its idiomatic meaning is easily inferred from the meaning of its constituent words. In contrast, the idiomatic meaning of the expression “*Estirar la pata*” (to die) is difficult to infer from its component words, making it a very opaque expression whose meaning has to be learned. Psycholinguistic research has revealed that decomposable idioms are processed faster than non-decomposable ones [11, 12]. Such advantage has been also observed in children. For instance, Caillies and Le Sourn-Bissaoui [13] showed that young children (5 year old) can easily comprehend decomposable idiomatic expressions in context, but it is not until the age of 7–8 that they are able to understand non-decomposable idioms. These findings have led some authors to suggest that different processes underlie the comprehension of decomposable and non-decomposable idioms [14]: Comprehending the former would involve the same processes of lexical retrieval and syntactic parsing as comprehending literal expressions. In contrast, non-decomposable idioms would be processed similarly as if they were single words [15].

Another relevant variable is final word predictability [5, 16, 17], which refers to the likelihood of providing a correct idiomatic completion in a fill-in-the-blank (cloze) task (e.g., *estirar la* ____ / *Kick the* ____). For example, the idiom “*verse de higos a brevas*” (e.g., to meet very rarely; literally: to meet from figs to figs) is fairly predictable in Spanish, given that an alternate completion is highly unlikely (e.g., *verse de higos a manzanas*; literally: to meet from figs to apples), and even semantically anomalous. However, the idiom “*hacerse el sueco*” (e.g., pretending not to understand; literally: to make the Swedish yourself), allows a variety of correct idiomatic completions (*hacerse el tonto*: *e*.*g*., *play dumb*, *hacerse el muerto*: *e*.*g*., *play dead*, etc.), and many other non-idiomatic endings. Several studies have shown the relevance of this variable. For instance, Cacciari and Tabossi [4] found that the figurative meaning of high predictable idioms was recovered earlier than the figurative meaning of low predictable ones. Furthermore, the literal meaning of the last word was activated for low predictable idioms, but not for high predictable ones.

Two relevant variables are also familiarity and knowledge. Familiarity (or subjective frequency) is commonly defined as how often a listener or reader encounters an idiomatic expression in its spoken or written form [17, 18]. Several studies have shown that sentences are comprehended faster and more accurately if they contain idioms that are familiar to the speakers rather than unfamiliar [5, 9, 19, 20]. A related variable is the knowledge of the figurative meaning [6, 8] or meaningfulness [5, 6, 16, 21]. It refers to how well an individual knows the meaning of an idiomatic expression and it has been shown to be related with online reading idiom comprehension times [21].

Many of the above studies have relied on a limited set of idiomatic expressions collected from different sources [1, 4, 22]. However, in order to conduct comprehensive research on idiom processing, normative data including large sets of items are needed. There are already several published idiom databases, which have been elaborated by asking people to rate a few hundred idiomatic expressions in some relevant variables. Concretely, there are normative data for English (e.g., [23] [320 idioms]; [16] [870 idioms]; [20] [245 idioms]; [5] [219 idioms]; [24] [100 idioms]; [17] [171 idioms]), French ([21] [305 idioms]; [25] [160 idioms]; [26] [300 idioms]), Italian ([8] [245 idioms]), German ([6] [619 idioms]), Chinese ([27] [350 idioms]), Bulgarian ([28] [90 idioms]) and Dutch ([29] [374 idioms]). As far as we know, there are no normative data for Spanish idioms. Thus, the main aim of this study is to fill this gap by providing psycholinguistic and affective norms for a large set of idiomatic expressions (1,252 idioms, the largest dataset so far).

Apart from the abovementioned variables, we also collected ratings for affective valence and arousal. In terms of linguistic stimuli, the emotional valence of a word or expression refers to the extent to which it is considered to be positive or negative. Arousal refers to the potential activation caused by a word or expression (from relaxing to highly arousing), regardless of whether it is negative or positive [30, 31]. In the field of figurative language, the role of emotional variables has hardly been investigated. Early studies [32] showed that participants prefer figurative to literal language when they express autobiographical emotional experiences orally. Similarly, two discourse analysis studies [33, 34] found that participants prefer idiomatic expressions when they have to express complaints, either to be empathetic or to appear more convincing. More recently, Bowes and Katz [35] have shown that the use of metaphors creates a greater sense of intimacy between interlocutors than the use of literal language and increases dexterity in activities that require emotions and mental states being attributed to others (i.e., theory of mind abilities). These findings have been supported by a series of recent brain imaging studies which have shown that the cortical structures associated with emotional processing are activated to a greater extent if participants read phrases containing metaphors than if they read their literal counterparts [36, 37]. Similar findings have been reported in the comparison of affectively valenced idiomatic expressions and literal expressions [38]. These findings point to the relevance of the study of affective properties in relation to figurative language in general and idiom processing in particular. However, to date, only one normative study conducted in German has included such type of ratings [6], finding that most idioms (around 68%) were considered as negative by the speakers and only a small number were considered as positive (31%).

### The current study

The objective of this study is to provide descriptive norms for the psycholinguistic and affective properties of a large set of Spanish idiomatic expressions and to explore the relationships between all these properties. We have collected ratings of familiarity, knowledge of the idiomatic meaning, decomposability, literality, predictability, valence and arousal.

This database might be very useful for the study of figurative language processing because it will provide researchers with a large pool from which to select their experimental stimuli. The usefulness of the dataset is evident if one considers the large number of Spanish speakers in the world (either as a first or as a second language). Hence, also those researchers interested in the processing of idiomatic expressions in bilinguals can rely on this database. In fact, there are already some studies on this topic, which have found that idiom processing in a non-native language is modulated by the same variables as in the native language (e.g., [39]), although bilinguals seem to process figurative meaning with greater difficulty than literal meaning [40].

Apart from research conducted with healthy speakers, in the last years there has been an increasing interest in the study of figurative language in several pathologies. For instance, the studies conducted with schizophrenic patients have shown that they prefer the literal meaning of idioms above their figurative meaning (e.g., [41, 42]). Other populations of interest have been patients with an autism spectrum disorder (e.g., [43, 44]; see [45] for an overview), aphasic patients [46] and Alzheimer patients [47]. A deficit in the processing of idiomatic expressions has been found in all these populations.

The aforementioned research is very relevant in order to characterize the deficits in the pragmatic use of language in those pathologies. Like in research conducted with healthy participants, the usual procedure to obtain the experimental materials has been first to select a number of idioms from different sources and second to refine the selection through different pilot studies. This is a laborious process, which highlights the importance of having normative databases. They enable researchers to select idioms with certain values in variables such as familiarity, literality, or decomposability, etc., according to the requirements of their particular study. Knowing the affective properties of the idioms (i.e., valence and arousal) is also of great value. The reason is that when there is a deficit in emotional processing, such variables are very relevant in order to assess language processing in this type of patients (e.g., schizophrenia or autism). Therefore, both basic and applied research can greatly benefit from the current dataset.

## Methods

### Participants

Participants (N = 965; 788 women) were students from four Spanish universities (Universitat Rovira i Virgili, Universidad Complutense de Madrid, Universidad Nacional de Educación a Distancia, Universidade de Santiago de Compostela) who agreed to complete the questionnaires to obtain course credits. They were all native speakers of Spanish, although in some cases they also spoke other official Spanish languages (Catalan or Galician). Their ages ranged between 16 and 58 years old (mean = 21.51; *SD* = 5.91; median = 20). All participants provided informed consent in accordance with the guidelines approved by the Ethics and Research Committees of the Rovira i Virgili University. In particular, the Ethics Committee for Research on People, Society and the Environment specifically approved this study (CEIPSA-2020-PR-0009). Written consent was obtained just before starting the survey. Participants were asked to read the full study information (participant information sheet) that appeared on the screen prior to the start of the questionnaire. By ticking a box, participants agreed to know the details of the study and consented to participate (with the possibility to withdraw at any time). If the acceptance box was not ticked, the questionnaire could not be started. Participants had the right to withdraw their consent at any time during the survey.

### Materials

The aim of this study was to collect a representative number of everyday idioms in Spanish. To this end, a total of 1,252 Spanish idiomatic expressions were selected from a variety of sources. The main source was a brainstorming carried out by the authors in different sessions. During these sessions, several specialized books on the compilation of idioms [48, 49] and websites (http://hispanoteca.eu/LexikonPhraseologie.asp; https://www.expresiones-espanolas.com/, etc.) were consulted. The problem with some compilations, however, is that they contain a large number of idioms that are in disuse. Only those we considered to be currently used by Spanish speakers were selected. We tried to draw up a set of heterogeneous idioms fulfilling a minimal structure of a verb phrase (VP) with one or more arguments. “*Estirar* (VP) *la pata* (Direct object)” (to die), or “*Poner* (VP) *verde* (Direct object) *a alguien* (Indirect object)” (put somebody down) are examples of this type of structure. Proverbs were discarded. Furthermore, for the sake of uniformity, although some idiomatic expressions could be inflected for person and time, we presented the idioms in the infinitive form whenever possible.

### Procedure

The total set of idiomatic expressions (1,252) was randomly divided over 12 questionnaires of approximately 104 idiomatic expressions each. Hence, there were 12 questionnaires for each variable (Familiarity, Knowledge, Decomposability, Literality, Predictability, Valence and Arousal), which were created with TestMaker [50]. Participants were recruited from the students of the subjects in which the authors teach. They were first asked to indicate their willingness to participate in the study. Once we obtained the list of participants willing to complete the questionnaires, between 1 and 3 questionnaires were randomly distributed to each participant. The participants received the links to the questionnaires by email, where they were also provided with some instructions and recommendations to complete them. They were instructed to fill in the questionnaires using a computer, not a mobile device, without the help of external resources, and to do it in a quiet environment with no distractions.

Each idiom was rated on each variable by an average of 20 participants. Every participant completed an average of two questionnaires on one or more variables. Each questionnaire began with some written instructions. In each case, the variable to be evaluated was defined and (when appropriate) the Likert scale was explained (i.e., extreme and middle points). The original Spanish instructions together with the English translation can be found in S1 Appendix (see also S2 Appendix for an example of a questionnaire used in the study). For *Familiarity*, participants were asked to rate the frequency with which they had heard, read or used the idiom, without taking into consideration if they knew its meaning. The rating scale ranged from 1 (*never heard/read/used*) to 7 (*often heard/read/used*). *Knowledge* of an idiom was defined as the extent to which the participant knows its meaning. The rating scale ranged from 1 (*I don’t know the meaning at all*) to 7 (*I know the meaning very well*). After this quantitative assessment, participants were asked to write down the idiomatic meaning of the expression in a text box. The authors checked the meanings provided by the participants to quantify the percentage of knowledge of each idiom (i.e., the percentage of participants who provided a correct definition of the meaning, *Knowledge_%)*. Of note, before coding the responses (meanings) provided to the Knowledge_% questionnaires, the five authors met to determine by consensus the figurative meaning of each idiom. To code a meaning as correct, we considered whether the response written by the participant conveyed the same meaning as the one previously defined by the five authors. Coding was first carried out individually by two of the authors, then these two authors met to compare their results and discuss discrepancies regarding the correctness of the responses given by the participants. Only those answers where there was consensus between the two authors were considered correct. For *Decomposability* (i.e., whether the figurative meaning of each idiomatic expression can be deduced from the meaning of the words it is made up of), participants were asked to indicate whether each idiom was decomposable or non-decomposable with a checkbox response. To facilitate the task, the figurative meaning of each expression was added in parentheses next to the idiom. *Predictability* refers to the likelihood of providing a correct idiomatic completion in a fill-in-the-blank (cloze) task. Participants were asked to complete the gap at the end of each sentence with the first word that came to mind. The authors assessed the correctness of the answer (by checking and correcting typos, spelling errors, grammatical gender incongruities, etc.) and calculated the percentage of correct completions. Only those answers that included the appropriate word were considered correct. *Literality* refers to the extent to which an idiomatic expression can be interpreted literally (i.e., plausibility of the interpretation). The rating scale for this variable ranged from 1 (*It has no plausible literal interpretation*) to 7 (*It has a clear and plausible literal interpretation*). *Emotional valence* refers to the extent to which an idiomatic expression is positive or negative. The rating scale ranged from 1 (*very negative*) to 7 (*very positive*), with 4 being the neutral point. Finally, *Emotional arousal* refers to the extent to which an idiomatic expression is arousing or exciting. The scale ranged from 1 (*not at all arousing*) to 7 (*very arousing*), with 4 being the neutral point. It should be noted that the questionnaires for the last three variables (literality, emotional valence and emotional arousal) contained an optional check box to indicate that the idiom or its figurative meaning was not known.

## Results and discussion

### Availability of the database

The database can be downloaded from the following link: https://osf.io/p75c3/. It is an Excel File, which includes the following columns: numerical_id (a numerical id that identifies each idiom), idiom (the text of the idiom), idiom_literal_translation (English translation of the idiom), idiomatic_meaning (a brief definition of the figurative meaning of the idiom), idiomatic_meaning_translation (English translation of the idiomatic meaning), familiarity (mean familiarity value of the idiom), knowledge (mean knowledge value of the idiom), knowledge_% (percentage of participants who provided the idiom’s correct meaning), decomposability (mean decomposability value of the idiom), literality (mean literality value of the idiom), predictability_% (percentage of participants who correctly completed the idiom in the predictability questionnaire), valence (mean valence value of the idiom), arousal (mean arousal value of the idiom), length_in_letters (number of letters of the idiom), length_in_words (number of words of the idiom). Next to each variable column there is a column (n_*variable_name*; e.g., n_familiarity) which states the number of responses that were used to calculate the mean score of such variable. Standard deviations for each variable are provided in the *sd_variable_name* columns (e.g., sd_familiarity). It should be noted that the ratings for unknown idioms were excluded from the calculation of the mean score for each variable and, consequently, some of the means were calculated on a lower number of responses.

### Data trimming

A total of 1,918 responses were given by 965 participants. Some of these responses were removed after a trimming procedure was applied. First, we removed the responses to questionnaires containing less than 50% of data. In addition, for each version of the questionnaires, we computed Pearson’s correlation coefficient between each participant’s values and the mean values of all participants who completed the same version. Then, we excluded the responses from those participants with negative correlation coefficients because the negative value suggests that they understood the scale in the opposite direction. We also removed the responses with correlation coefficients near to zero (i.e., less than or equal to .15), because this would suggest that the participant responded randomly to the questionnaire. By using this trimming procedure, we removed 39 responses.

### Reliability

We calculated the mean intraclass correlation coefficients (ICCs) of the questionnaires to assess the interrater reliability of the variables. We excluded predictability and knowledge_%, since the values of these questionnaires were corrected by the authors. All variables showed a good to excellent reliability (see [51] for interpretation guidelines): .94 for familiarity, .90 for knowledge, .85 for decomposability, .91 for literality, .61 for valence, and .93 for arousal.

### Descriptive statistics

A summary of the descriptive statistics of the 1,252 idioms in the database is provided in Table 1.

**Table 1:** Descriptive statistics of the variables in the database.

Variable	Mean	*SD*	Minimum	Maximum	Mean Valid Responses	Skewness
**Familiarity**	4.47	1.43	1.15	6.90	24.27	-0.48
**Knowledge**	5.29	1.45	1.11	7.00	19.75	-0.96
**Knowledge_%**	70.88	28.87	0.00	100	20.00	-0.86
**Decomposability**	0.47	0.28	0.00	1.00	24.33	0.13
**Literality**	3.96	1.27	1.08	6.64	20.56	0.10
**Predictability_%**	36.96	31.91	0.00	100	19.79	0.44
**Valence**	3.41	1.48	1.05	6.96	20.64	0.53
**Arousal**	3.76	0.92	1.68	6.62	21.06	0.24
**Length in words**	4.18	1.49	2.00	11	-	0.78

Note. The data from the variables *Knowledge_%* and *Predictability_%* are given in percentages. The mean of *Decomposability* is based on participants’ binary selections (0 = non decomposable or 1 = decomposable) for each item.

The data distributions for familiarity and knowledge are clearly negatively skewed (right bias). The average values observed for both variables indicate that most participants know the set of idioms well and are quite familiar with them. The decomposability rating in Table 1 is the average of participants’ choice between the non-decomposable (0) and decomposable (1) options. It can be observed that the distribution of this variable is slightly biased towards the left (positive skew), although an inspection of the data showed that approximately half of the idioms were rated as non-decomposable and the other half as decomposable. The distribution of the literality variable is highly symmetric, and the average rating indicates that the idioms that have a plausible literal interpretation do not stand out over those that do not. Predictability data are clearly positively skewed. Furthermore, as can be seen in Table 1, the average number of correct completions was not very high. This is because in such a broad set of items, there are many short idioms whose final word is highly unpredictable, e.g., *estar en las nubes* (to be in the clouds), *estar en la calle* (to be in the street), *estar en las últimas* (to be on your last legs), etc. The distribution of the valence data is clearly positively skewed, indicating that there is a higher proportion of negative than positive idioms. Finally, the distribution of arousal data is also slightly positively skewed. This suggests that the database contains more low than high arousing idioms.

### Correlations between variables

We calculated the partial correlations between all the variables. Table 2 shows Pearson’s correlation coefficients between each pair of variables after controlling for the other ones. To show just one example, the correlation between familiarity and knowledge (*r* = .61) represents the correlation coefficient between these two variables after controlling for the correlation of these two variables with knowledge_%, decomposability, literality, predictability, valence, arousal and length in words. First, we describe the correlations between psycholinguistic variables (i.e., familiarity, knowledge, decomposability, literality, predictability, and length in words). Then, we describe the correlations between psycholinguistic and affective variables (i.e., valence and arousal). Only those correlations that reached a significance level of *p <* .05 are discussed.

**Table 2:** Partial correlations between the variables in the database.

	Familiarity	Knowledge	Knowledge_%	Decomposability	Literality	Predictability	Valence	Arousal	Length in words
**Familiarity**	-	.61***	ns	ns	ns	.26***	ns	ns	-.20***
**Knowledge**		-	.66***	.09**	ns	ns	-.07*	.10***	ns
**Knowledge_%**			-	.15***	-.07*	ns	ns	ns	ns
**Decomposability**				-	.25***	-.08**	.08**	ns	.16***
**Literality**					-	.14***	.07*	ns	-.10**
**Predictability**						-	ns	ns	.54***
**Valence**							-	.40***	ns
**Arousal**								-	ns
**Length in words**									-

Note. ns: non significant correlation

* p < .05

** p < .01

*** p < .001.

#### Correlations between psycholinguistic variables

The first result worth pointing out is the strong positive correlation between familiarity and the extent to which participants consider that they know the meaning of the idiom (knowledge). Such result is in line with other normative studies that have found moderate or strong positive correlations between these two variables [16, 21, 25, 27], and suggests that the more familiar an idiom is rated, the more confident the speakers are in their knowledge of its meaning. However, in contrast to prior research [6, 8, 29], no correlation was found here between familiarity and the actual knowledge of the idiom meaning (knowledge_%, the percentage of participants who provided a correct definition of the meaning). This result indicates that the familiarity of an idiom is more related with a subjective feeling of knowing its meaning than with actually knowing it.

Familiarity also showed a small negative correlation with length in words (see [6, 25] for similar results), and a small positive correlation with predictability (see [5, 8, 16, 21, 25, 27] for a similar pattern of findings); so that, the more familiar an idiom is rated, the shorter and more predictable it tends to be. It should be mentioned, however, that there are also some exceptions, in which non-familiar idioms are highly predictable (e.g., *abrir el paraguas antes que llueva*; literally: open the umbrella before it rains). In this case, however, length seems to play an important role, because longer idioms, although being unfamiliar, can be completed only by a restricted number of words. This is reflected here by the moderate positive correlation found between predictability and length in words (also reported in [8, 21, 25]).

Concerning the knowledge of the meaning of the idioms, the two measures collected here (knowledge and knowledge_%) showed a high positive correlation. This indicates that the speakers’ intuitions with respect to their knowledge are indeed reliable. There was also a small positive correlation between both measures of knowledge and decomposability. This pattern of findings, which was also observed in other studies [5, 8, 16, 21, 25, 27, 29], suggests that the more decomposable (easier to deduce the figurative meaning from its constituent words) an idiom is rated, the higher the percentage of participants who know its meaning, as well as their confidence in such knowledge. Furthermore, in line with Libben and Titone’s study [5], there was a small negative correlation between the actual knowledge (knowledge_%) and literality, indicating that the meaning of unambiguous idioms (i.e., those that can only be interpreted in their figurative form) tends to be better known than that of ambiguous ones (i.e., those that can be interpreted in both their literal and figurative forms).

Finally, decomposability showed a small positive correlation with length in words (see [6] for a similar result), a moderate positive correlation with literality (as in [27]), and a small negative correlation with predictability (contrary to [21, 27], who observed a positive correlation between these variables). These results indicate that those idioms whose figurative meaning is easier to deduce from their constituent words tend to be more ambiguous, longer, and less predictable. A prototypical idiom to exemplify these correlations would be “Ser más viejo que un camino” the literal translation of which is “To be older than a road”, as it scores high on decomposability and literality, low on predictability and is long. In addition, literality revealed a small positive correlation with predictability (see [27] for a similar result), and a small negative correlation with length in words, which suggests that ambiguous idioms tend to be more predictable and shorter than unambiguous idioms. A prototypical example here would be “Darlo todo”, the literal translation of which is “Give it all”, as it scores high on literality and predictability and is short.

#### Correlations between psycholinguistic and affective variables

**Fig 1:**
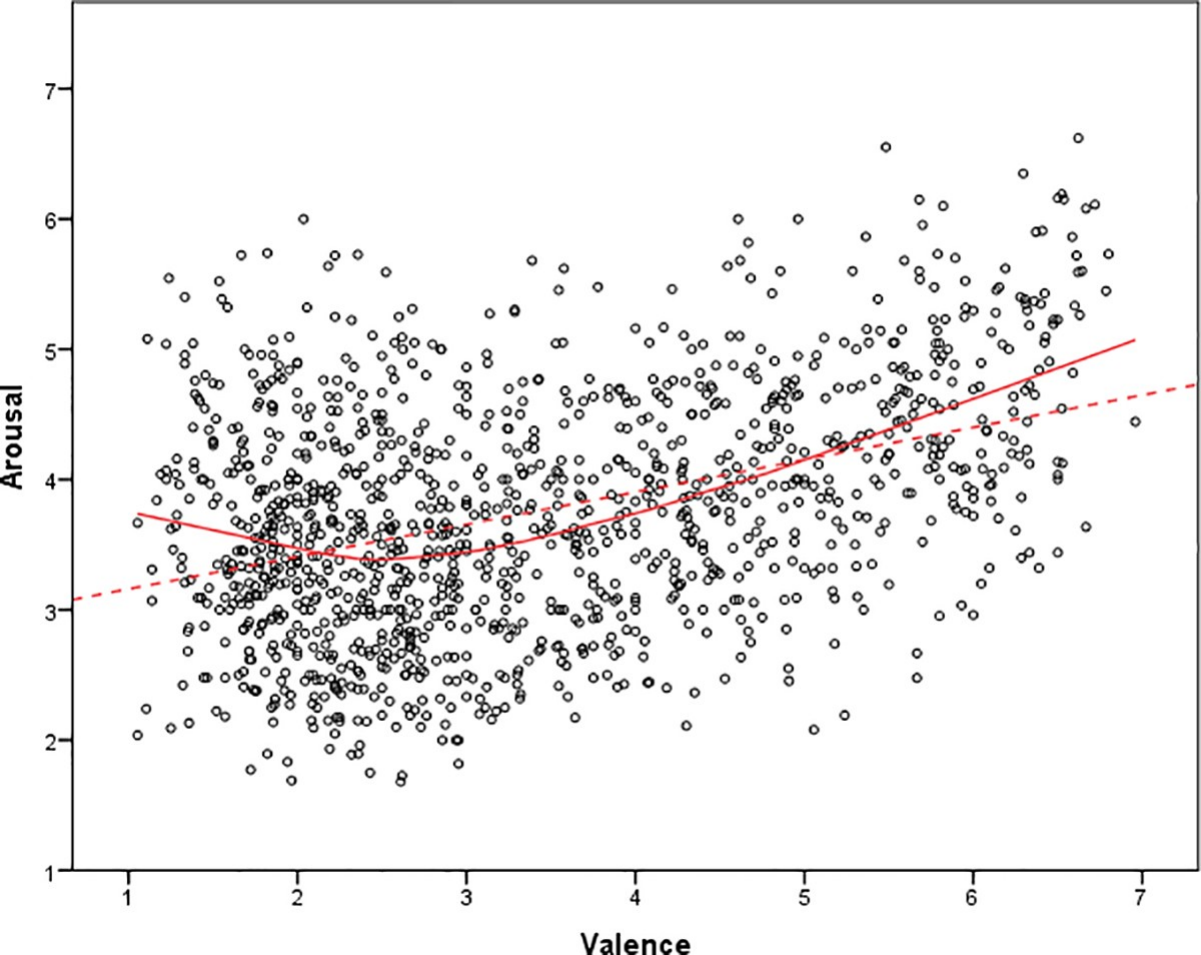
Valence ratings plotted against arousal ratings. Each dot represents the mean for each word across participants.

For the affective variables, first we classified the idioms into positive and negative, an approach similar to that used by Citron et al. [6] who rated their idioms on a scale of -3 to +3. They considered as negative idioms those with values below 0, and positive idioms those with values above 0. Since we have used a scale from 1 to 7, we have considered ratings below 4 as indexing negative valence and ratings above 4 as indexing positive valence. With this criterion, 826 idioms (66% of the database) would be negative, 415 (33%) would be positive, and only 11 had a score of exactly 4. This distribution is very similar to that obtained by Citron et al. [6] with German idioms. Since there has been no a priori bias in collecting the sample of idioms, this would mean that when we express ourselves idiomatically in conventional speech, Spanish has more negative than positive expressions. Alternatively, we can relate this finding to the evaluative function of figurative language and may indicate that idiomatic expressions as an indirect form of communication are preferred to literal language when speakers make negative statements [33]. Second, we examined arousal values to classify the idioms into low arousing (values below 4) and high arousing (values above 4). Following this criterion, 754 idioms (60%) would be low arousing, 467 (37%) would be high arousing, and 31 idioms had a score of exactly 4. Apart from examining the distribution of idioms in the dataset according to their affective properties, we calculated the correlations between affective variables, and found a moderate positive correlation between valence and arousal (*r* = .40). Valence and arousal ratings were plotted against each other and the results showed a quadratic relationship (Fig 1). This is in line with previous results with idioms [6] and isolated words (e.g., [52, 53]), suggesting that the more valenced an idiom is rated (either positively or negatively but, in this study, mainly positive), the more arousing it is judged.

Finally, we examined the relationship between affective and psycholinguistic variables and found overall low or very low correlations. Indeed, the speakers’ confidence in their knowledge of the idioms’ meaning showed a small negative correlation with valence and a small positive correlation with arousal. This indicates that such confidence is higher for the more negative and arousing idioms. However, there was not any correlation between affective variables and the actual knowledge. In addition, valence showed a small positive correlation with decomposability, indicating that the meaning of positive idioms tends to be easier to deduce from their constituent words than the meaning of negative ones. Finally, there was a small positive correlation between valence and literality, which suggests that the idioms rated as more positive in the database tend to be judged as more ambiguous than the negative ones.

These findings do not mimic the ones reported by Citron et al. [6], who found a positive correlation between valence and familiarity and between arousal and both familiarity and semantic transparency (relatively similar to our decomposability). They also reported a positive correlation between arousal and figurativeness (which is the inverse of literality). Although there is no a clear explanation for the differences in the pattern of results, they may be related with the size of the dataset, which is almost double in our study as in the one by Citron and co-workers. Further research in the field is needed to study the relationship between affective and psycholinguistic variables in figurative language processing.

Lastly, it should be noted that each idiom was rated by an average of 20 participants. This average is slightly below those used in some similar studies, although it is a common average in normative databases of words in isolation. Considering that there is a clear convergence between our results and the results of previous studies, we do not consider this fact to have affected the results. However, we would like to point this out as a possible limitation of the study.

## Conclusions

This study reports normative data on psycholinguistic (familiarity, knowledge, decomposability, literality and predictability) and affective (valence and arousal) properties of a large set of Spanish idioms. It also explores the interrelationships between all these variables. The results reveal a pattern of relationships between psycholinguistic variables that is similar to those found in previous studies conducted in other languages. As far as affective properties are concerned, the common inverted U-shaped relationship between valence and arousal is observed. As for the hedonic tone of the idioms in the database, most of them (66%) are perceived as negative by the speakers and low arousing (60%). This database, the first one drawn up in Spanish, complements normative studies already available in other languages. It will be of great help for researchers interested in the representation and processing of figurative language in Spanish, both in normal and clinical populations. The internal consistency indicated by the high reliability indices makes it a powerful tool for the experimental design of basic and applied psycholinguistic studies in the field.

## Supporting information

S1 Appendix Instructions for the online rating of Spanish idioms. (DOCX)

S2 Appendix Example of a questionnaire used in the study to assess arousal. (DOCX)
