# Supplementary material for: Psycholinguistic and affective norms for 1,252 Spanish idiomatic expressions
Source: PLoS One. 2021 Jul 16;16(7):e0254484. doi: 10.1371/journal.pone.0254484 (PMC8284670; doi:10.1371/journal.pone.0254484)
Supplement: S1 Appendix — (DOCX) [file pone.0254484.s001.docx]

**S1 Appendix. Instructions for the online rating of Spanish idioms**

Note: The instructions in Spanish come first, as seen by the participants and then the English translation. The idioms used as examples in Spanish have not been translated into English.

**Instrucciones generales**

A continuación vas a completar un cuestionario en el que se te presentarán frases hechas (también conocidas como “modismos”). Estas frases poseen un significado figurado (metafórico) que no se deriva directamente del significado literal de las palabras que las componen. Por ejemplo, la frase hecha “saber al dedillo” tiene un significado figurado (“conocer algo muy bien”) que no puede derivarse del significado literal de sus palabras. Queremos que evalúes algunas características específicas de las frases hechas que encontrarás a continuación. Sigue las instrucciones que te especificamos al inicio de la tarea. ¡Muchas gracias!

**Instrucciones específicas**

*Familiaridad*

Por favor califica la FRECUENCIA con la que has escuchado, leído o utilizado cada una de las siguientes frases. Por ejemplo, la frase “echarlo a cara o cruz”, se utiliza con mucha frecuencia, mientras que la frase “comer como un sabañón”, casi nunca se usa. La escala para la valoración de FRECUENCIA va de 1 a 7, donde 1 significa NUNCA HE ESCUCHADO/LEÍDO/UTILIZADO ESTA EXPRESIÓN y 7 A MENUDO ESCUCHO/LEO/UTILIZO ESTA EXPRESIÓN. Una puntuación de 4 indicaría que “algunas veces escucho/leo/utilizo esta expresión”.

*Conocimiento del significado idiomático*

Por favor evalúa EN QUÉ MEDIDA CONOCES EL SIGNIFICADO FIGURADO (METAFÓRICO) de cada una de las siguientes frases hechas. Por ejemplo, la frase “enseñar la puerta”, en términos metafóricos o figurados, significa “despedir a alguien”, mientras que en términos literales significa “mostrar a alguien una puerta”. Lo que te pedimos es que evalúes sólo cuánto conoces el significado figurado, no el literal. La escala de valoración de CONOCIMIENTO va de 1 a 7, donde 1 significa NO CONOZCO EL SIGNIFICADO FIGURADO EN ABSOLUTO, y 7 significa CONOZCO EL SIGNIFICADO FIGURADO MUY BIEN. Una calificación de 4 significa “Conozco el significado figurado sólo aproximadamente”. Justo después de cada evaluación, por favor ESCRIBE brevemente el significado figurado de la frase.

*Descomponibilidad*

En esta tarea se te pide que juzgues si el SIGNIFICADO FIGURADO (METAFÓRICO) de cada frase hecha se puede DEDUCIR DEL SIGNIFICADO LITERAL de las palabras que la componen. Por ejemplo, el significado figurado de la frase “enseñar la puerta” (despedir a alguien) se puede deducir del significado literal de sus palabras. Un ejemplo similar sería “no oírse ni una mosca” (no hay ruido). Por el contrario, el significado figurado de la frase “poner los cuernos” (ser infiel a tu pareja) no puede llegar a deducirse del significado literal de sus palabras. A continuación verás un conjunto de frases hechas junto a su significado literal (presentado entre paréntesis). En cada frase debes indicar si su significado figurado se puede deducir del significado literal de cada una de sus palabras. Siguiendo con los ejemplos anteriores, marcarías la casilla “SÍ” para la frase “no oírse ni una mosca”, y la casilla “NO” para la frase “poner los cuernos”.

*Literalidad*

Para cada frase del cuestionario tendrás que hacer un juicio de LITERALIDAD. Mientras que todas las frases hechas tienen una interpretación metafórica o figurada clara, sólo algunas tienen un significado literal claro y con sentido. Por ejemplo, la frase “enseñar la puerta”, en términos metafóricos o figurados, significa “despedir a alguien”, mientras que en términos literales significa “mostrar a alguien una puerta”. Sin embargo, la frase “pagar a toca teja”, en términos figurados significa “pagar al contado/inmediatamente”, pero en términos literales no tiene un significado claro si lo comparamos con “enseñar la puerta”, ya que es difícil pensar en qué significa literalmente “pagar a toca teja”. Tu tarea al valorar las frases del cuestionario es decidir si la frase en cuestión tiene una interpretación literal posible, y si es así, cómo es de plausible en una escala de 1 a 7. Una puntuación de 1 indicaría que la frase evaluada NO TIENE UNA INTERPRETACIÓN LITERAL PLAUSIBLE, mientras que una puntuación de 7 indicaría que TIENE UN SIGNIFICADO LITERAL CLARO Y PLAUSIBLE.

*Predictibilidad*

En este cuestionario leerás una serie de frases hechas que tienen un hueco en el que falta una palabra. Debes escribir en la caja de texto habilitada, la primera palabra que te venga a la mente para completar la frase. Por ejemplo, si te presentan la frase incompleta “La cabra tira al …………”, la primera palabra en venir a la mente podría ser “monte”. Si así fuese, escribirías la palabra “monte” en el espacio previsto. Debes utilizar una única palabra en cada caso.

*Valencia*

La VALENCIA EMOCIONAL describe la medida en que un acontecimiento o situación es positivo o negativo. Por ejemplo, la frase “podrido hasta la médula” describe algo negativo, mientras que la frase “ser el amo” describe algo positivo. Tu tarea en este caso es evaluar cómo es de positivo o negativo el significado metafórico o figurado de las frases hechas de este cuestionario. Una puntuación de 1 indica que la frase describe algo “MUY NEGATIVO”, mientras que una puntuación de 7 indica que la frase en cuestión describe algo “MUY POSITIVO”. Un valor intermedio (por ejemplo 4) indicaría que la frase describe algo que no es ni positivo ni negativo, como, por ejemplo, “cada cosa a su tiempo”.

*Arousal*

La INTENSIDAD EMOCIONAL describe hasta qué punto un evento es activador en términos emocionales. Por ejemplo, la frase “una noticia bomba” se percibe como muy activadora en términos emocionales, mientras que la frase “estar pachucho” se percibe como poco activadora. Por favor, evalúa cómo de activadores son los significados (metafóricos o figurados) de las siguientes frases hechas en la escala proporcionada. Una puntuación de 1 significa que la frase NO ES ACTIVADORA EN ABSOLUTO EN TÉRMINOS EMOCIONALES, mientras que una puntuación de 7 significa que la frase ES MUY ACTIVADORA. Una puntuación intermedia (4) significa que la frase es bastante activadora.

**Instructions for the online rating of Spanish idioms (English translation)**

**General instructions**

You are going to complete a questionnaire in which you will be presented with a set of phrases (also known as “idioms”). These phrases have a figurative (metaphorical) meaning that is not directly derived from the literal meaning of the words that make them up. For example, the idiom “saber al dedillo” has a figurative meaning (“know something very well”) that cannot be derived from the literal meaning of its words. We want you to evaluate some specific characteristics of the phrases that you will find below. Follow the instructions that we give you at the beginning of each task. Thank you very much!

**Specific instructions**

*Familiarity*

Please rate the FREQUENCY with which you have heard, read or used each of the following phrases. For example, the phrase “echarlo a cara o cruz” is used very often, while the phrase “comer como un sabañón” is almost never used. The scale for rating FREQUENCY goes from 1 to 7, where 1 means I HAVE NEVER HEARD/READ/USED THIS EXPRESSION and 7 means I HAVE HEARD/READ/USED THIS EXPRESSION very often. A score of 4 indicates that “I sometimes listen/read/use this expression”.

*Knowledge of the idiomatic meaning*

Please evaluate TO WHAT EXTENT YOU KNOW THE FIGURATIVE MEANING (METAPHORIC) of each of the following phrases. For example, the phrase “enseñar la puerta”, in metaphorical or figurative terms, means “to dismiss someone”, while in literal terms it means “to show someone the door”. We ask you to evaluate only the extent to which you know the figurative meaning, not the literal one. The KNOWLEDGE rating scale goes from 1 to 7, where 1 means I DO NOT KNOW THE FIGURATIVE MEANING AT ALL, and 7 means I KNOW THE FIGURATIVE MEANING VERY WELL. A score of 4 means “I know the figurative meaning only approximately”. Just after each evaluation, please WRITE down a short explanation of the figurative meaning of the sentence.

*Decomposability*

In this task we ask you to judge whether the FIGURATIVE (METAPHORIC) MEANING of each phrase can be DEDUCED from the LITERAL MEANING of the words that make it up. For example, the figurative meaning of the phrase “enseñar la puerta” (to dismiss someone) can be deduced from the literal meaning of its words. A similar example would be “no oírse ni una mosca” (to hear a pin drop). In contrast, the figurative meaning of the phrase “poner los cuernos” (to cheat on your partner) cannot be deduced from the literal meaning of his words. Below you will see a set of idioms together with their literal meaning (presented in brackets). For each idiom you must indicate whether its figurative meaning can be deduced from the literal meaning of each of the words. For the examples above, you would check the box “YES” for the phrase “no oírse ni una mosca” and the box “NO” for the phrase “poner los cuernos”.

*Literality*

You will have to judge the LITERALITY of each sentence. While all idioms have a clear metaphorical or figurative interpretation, only some have a clear and meaningful literal meaning. For example, the phrase “enseñar la puerta”, in metaphorical or figurative terms, means “to dismiss someone”, while in literal terms it means “to show someone the door”. However, the phrase “pagar a tocateja”, in figurative terms means “pay in cash/immediately”, but in literal terms it has no clear meaning when compared to “enseñar la puerta”, as it is difficult to think of what “pagar a tocateja” literally means. Your task is to decide whether the sentence in question has a possible literal interpretation, and if so, how plausible it is on a scale of 1 to 7. A score of 1 indicates that the sentence evaluated has NO PLAUSIBLE LITERAL INTERPRETATION, while a score of 7 indicates that it has a CLEAR AND PLAUSIBLE LITERAL MEANING.

*Predictability*

In this questionnaire, you will read a series of phrases that have a gap where one word is missing. You must write in the text box the first word that comes to mind to complete the sentence. For example, if you are presented with the incomplete sentence “La cabra tira al ...........”, the first word that comes to mind could be *“monte”*. If so, you would write the word *“monte”* in the space provided. You must use only one word in each case.

*Valence*

EMOTIONAL VALENCE describes the extent to which an event or situation is positive or negative. For example, the phrase “podrido hasta la médula” describes something negative, while the phrase “ser el amo” describes something positive. Your task here is to evaluate how positive or negative the metaphorical or figurative meaning of the idioms in this questionnaire is. A score of 1 indicates that the phrase describes something “VERY NEGATIVE”, while a score of 7 indicates that the phrase in question describes something “VERY POSITIVE”. An intermediate value (e.g. 4) indicates that the phrase describes something that is neither positive nor negative, such as “everything in its time”.

*Arousal*

EMOTIONAL INTENSITY describes the extent to which an event is activating or exciting in emotional terms. For example, the phrase “una noticia bomba” is perceived to be very activating, while the phrase “estar pachucho” is perceived to be not very activating. Please evaluate how activating the meanings (metaphorical or figurative) of the following phrases are on the scale provided. A score of 1 means that the phrase IS NOT ACTIVATING AT ALL, while a score of 7 means that the phrase IS VERY ACTIVATING. A score in the middle (4) means that the phrase is quite activating.
