# Supplementary figures and images for: Psycholinguistic and affective norms for 1,252 Spanish idiomatic expressions
Source: PLoS One. 2021 Jul 16;16(7):e0254484. doi: 10.1371/journal.pone.0254484 (PMC8284670; doi:10.1371/journal.pone.0254484)

**S2 Appendix. Example of a questionnaire used in the study to assess arousal.**
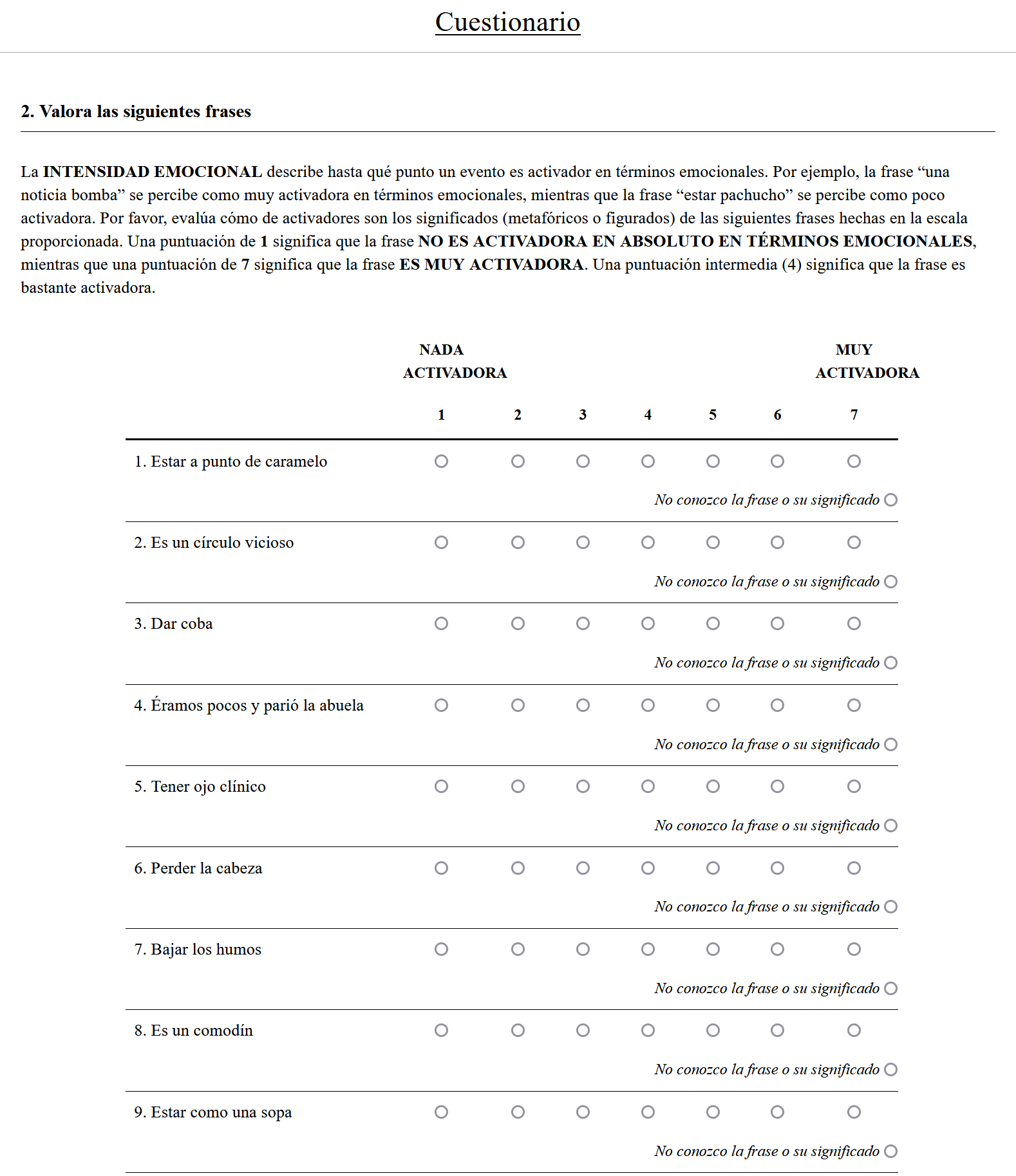

Supplement: S2 Appendix — (DOCX) [file pone.0254484.s002.docx]
